# Reappraisal of the prognostic value of Epstein-Barr virus status in monomorphic post-transplantation lymphoproliferative disorders–diffuse large B-cell lymphoma

**DOI:** 10.1038/s41598-021-82534-4

**Published:** 2021-02-03

**Authors:** Jwa Hoon Kim, Hyungwoo Cho, Heungsup Sung, Ah Ra Jung, Yoon Sei Lee, Sang-wook Lee, Jin-Sook Ryu, Eun Jin Chae, Kyoung Won Kim, Jooryung Huh, Chan-Sik Park, Dok Hyun Yoon, Cheolwon Suh

**Affiliations:** 1 Department of Oncology, Asan Medical Center, University of Ulsan College of Medicine, 88 Olympic-ro 43-gil, Songpa-gu, Seoul, 05505 Republic of Korea; 2 Department of Laboratory Medicine, Asan Medical Center, University of Ulsan College of Medicine, Seoul, Republic of Korea; 3 Department of Otolaryngology, Asan Medical Center, University of Ulsan College of Medicine, Seoul, Republic of Korea; 4 Department of Radiation Oncology, Asan Medical Center, University of Ulsan College of Medicine, Seoul, Republic of Korea; 5 Department of Nuclear Medicine, Asan Medical Center, University of Ulsan College of Medicine, Seoul, Republic of Korea; 6 Department of Radiology, Asan Medical Center, University of Ulsan College of Medicine, Seoul, Republic of Korea; 7 Department of Pathology, Asan Medical Center, University of Ulsan College of Medicine, Seoul, Republic of Korea; 8 Division of Oncology/Hematology, Department of Internal Medicine, Korea University Anam Hospital, Korea University College of Medicine, Seoul, Republic of Korea

**Keywords:** Cancer, Oncology

## Abstract

The role of the Epstein-Barr virus (EBV) status in the blood for predicting survival in post-transplantation lymphoproliferative disorders–diffuse large B-cell lymphoma (PTLD–DLBCL) is unknown. We evaluated the prognostic values of pre-treatment EBV-encoded small RNA (EBER) detected with in situ hybridization in tissues and EBV DNA in the whole blood (WB) and plasma in 58 patients with monomorphic PTLD–DLBCL after solid organ transplantation. There were no significant differences in the rates of overall response, complete response, and survival according to EBER EBV and WB EBV status. In contrast, patients with positive plasma EBV DNA had significantly lower rates of overall response (60.0% vs. 94.4%, *P* = 0.043) and complete response (40.0% vs. 88.9%, *P* = 0.019) as well as worse progression-free survival (PFS) (*P* = 0.035) and overall survival (OS) (*P* = 0.039) compared with patients with negative plasma EBV DNA. In multivariate analysis, plasma EBV DNA positivity was a significantly unfavorable prognostic factor for PFS [hazard ratio (HR) 4.92, 95% confidence interval (CI) 1.22–19.86, *P* = 0.025] and OS (HR 4.48, 95% CI 1.14–17.63, *P* = 0.032). Despite small number of 6 patients with plasma EBV positivity, plasma EBV DNA positivity might be more prognostic for survival than EBER or WB EBV DNA positivity in patients with monomorphic PTLD–DLBCL.

## Introduction

Post-transplantation lymphoproliferative disorder (PTLD), which refers to newly developed lymphoid or plasmacytic proliferation after transplantation^1,2^, is a major concern in patients undergoing solid organ transplantation^3,4^. The incidence of PTLD has been reported to be as high as 20% depending on the type of transplantation, multiple organ transplantations, and the intensity or the type of immunosuppressive agents used^3–5^. PTLD occurs commonly in heart (1%–2%), heart/lung (> 5%), liver (1%–2%), and kidney (< 1%) transplant recipients^2,3,5^. PTLD has a wide spectrum that range from early lesion and polymorphic lymphoproliferation to monomorphic lymphoma^6^. PTLD also has various histologic subtypes, of which diffuse large B-cell lymphoma (DLBCL) is the most common subtype^7^.

Epstein-Barr virus (EBV) plays a crucial role in the development of PTLD, accounting for 60% to 85% of PTLD cases^2,8^. Primary EBV infection is more common in children, and EBV causes lifelong infection in approximately 90% of adults with seropositive status^9,10^. Impaired immune surveillance due to the use of immunosuppressive agents following transplantation leads to lymphoproliferation driven by latent EBV infection^8,11^. Therefore, the evaluation of EBV status by EBV-encoded small RNA (EBER) in situ hybridization (ISH) of the primary tumor is recommended for the diagnosis of PTLD, and PTLD patients are classified as EBV (+) or EBV (−) based on these results. Regarding the prognostic value of EBV status in PTLD, previous studies reported discordant results on the effect of EBV status on survival in PTLD patients based on EBER-ISH of the primary tumor^11–16^. However, most of the previous studies included patients with heterogeneous histologic subtypes treated with various treatment approaches, which may have contributed to such conflicting results.

The quantification of EBV DNA in the blood is non-invasive and more convenient than EBER-ISH, and the blood EBV status was shown to be able to predict survival outcomes in several EBV-associated diseases including lymphomas^17^. Specifically, the blood EBV status in patients with Hodgkin or non-Hodgkin lymphoma such as T/natural killer cell lymphoma has shown a strong association with prognosis^18–22^. However, the prognostic role of the blood EBV status in PTLD has not been investigated, and there is no consensus regarding the optimal type of blood sample (i.e., whole blood [WB] or plasma) for the measurement of EBV DNA.

Here, we compared the prognostic values of EBER, WB EBV DNA, and plasma EBV DNA in a homogeneous group of patients with monomorphic PTLD–DLBCL after solid organ transplantation who were treated with chemotherapy.

## Results

### Patients and treatment

**Figure 1:**
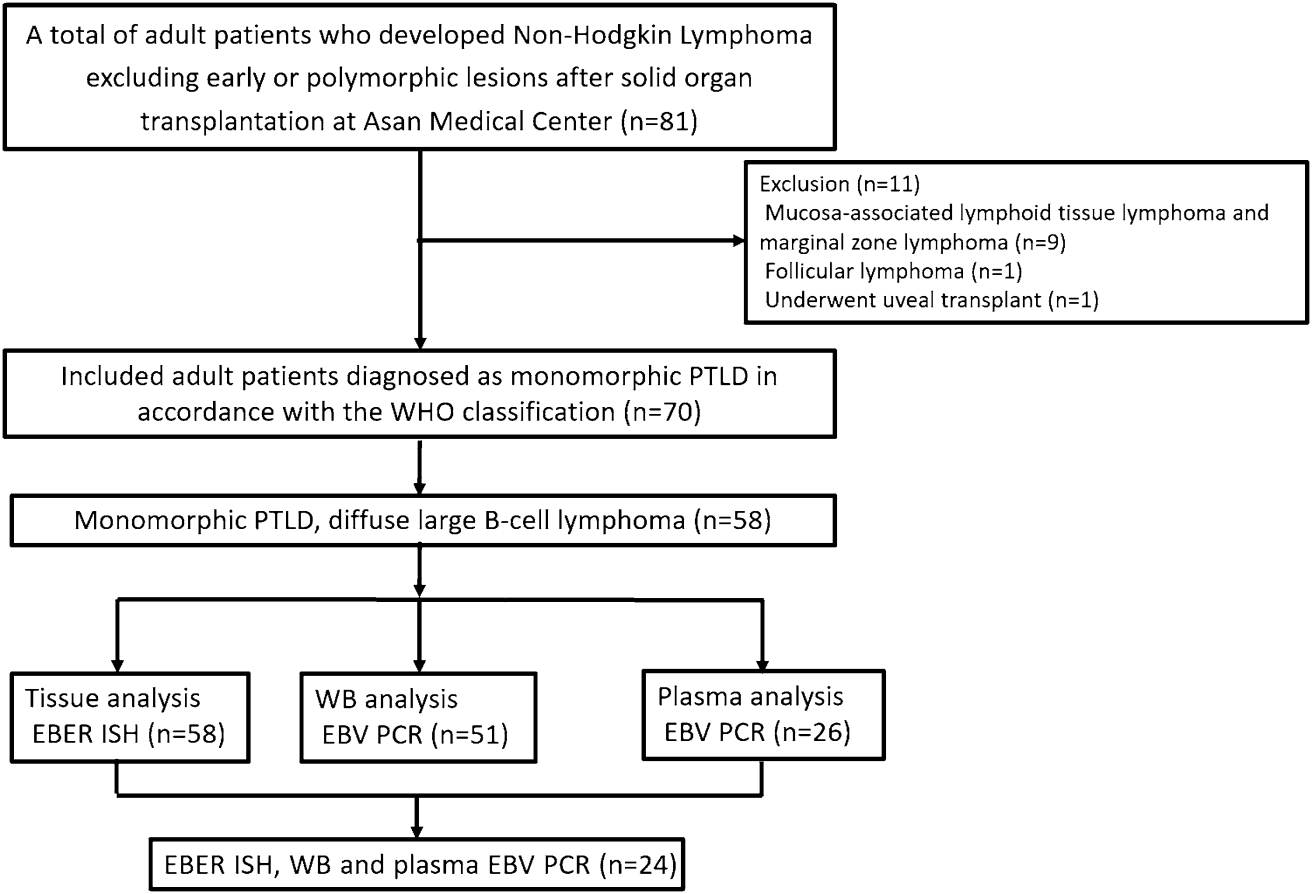
Consort diagram. *PTLD* post-transplantation lymphoproliferative disorder, *WHO* world health organization, *EBER* Epstein-Barr virus-encoded small ribonucleic acids, *ISH* in situ hybridization, *WB* whole blood, *EBV* Epstein-Barr virus, *PCR* polymerase chain reaction.

A total of 70 adult patients with new onset monomorphic PTLD after solid organ transplantation were diagnosed at Asan Medical Center (Seoul, South Korea) between December 2004 and June 2019 (Fig. 1); of them, 58 patients with monomorphic PTLD–DLBCL were included in the analysis. Table 1 summarizes the baseline characteristics of the patients according to the pre-treatment status of EBER, WB EBV, and plasma EBV, which were assessed in all the available samples (Fig. 1 and Supplementary Fig. 1). In the whole cohort, the median age at diagnosis was 55 years (range 19–75) and 39 (67.2%) patients were male. At the time of transplantation, 41 (70.7%) patients were EBV seropositive and one (1.7%) patient was EBV seronegative; the EBV status was unknown or not available in 16 (27.6%) patients who had undergone transplantation at other hospitals. The median time from transplant to the diagnosis of PTLD was 5.5 years (range 0.1–29.5); 13.8% had early-onset PTLD (< 1 year) and 86.2% had late-onset PTLD ($\ge$ 1 year). Thirty-eight (65.5%) patients had stage III–IV diseases, and 11 (19.0%) patients had an international prognostic index (IPI) score^23^ of 4–5. Most of the patients (n = 57, 98.3%) were treated with chemotherapy and one patient underwent surgical resection without any chemotherapy.

**Table 1:** Baseline characteristics of the patients according to the pre-treatment EBER, whole blood EBV, and plasma EBV status.

		EBER (n = 58)			WB EBV (n = 51)			Plasma EBV (n = 26)	
	Pre-treatment EBER (n = 58, 100%)	EBER (+) (n = 19, 32.8%)	EBER (−) (n = 39, 67.2%)	Pre-treatment WB (n = 51, 100%)	WB EBV (+) (n = 22, 43.1%)	WB EBV (−) (n = 29, 56.9%)	Pre-treatment plasma (n = 26, 100%)	Plasma EBV (+) (n = 6, 23.1%)	Plasma EBV (−) (n = 20, 76.9%)
Age at diagnosis, median (range)	55 (19–75)	51 (19–75)	56 (22–73)	55 (19–73)	54 (19–68)	55 (21–73)	51 (19–68)	62 (19–68)	50 (21–68)
**Sex**									
Male	39 (100)	13 (33.3)	26 (66.7)	35 (100)	15 (42.9)	20 (57.1)	16 (100)	2 (12.5)	14 (87.5)
Female	19 (100)	6 (31.6)	13 (68.4)	16 (100)	7 (43.8)	9 (56.2)	10 (100)	4 (40.0)	6 (60.0)
**Transplant type**									
Kidney	22 (100)	7 (31.8)	15 (68.2)	19 (100)	6 (31.6)	13 (68.4)	11 (100)	1 (9.1)	10 (90.9)
Liver	27 (100)	7 (25.9)	20 (74.1)	24 (100)	10 (41.7)	14 (58.3)	10 (100)	1 (10.0)	9 (90.0)
Heart	7 (100)	4 (57.1)	3 (42.9)	6 (100)	5 (83.3)	1 (16.7)	5 (100)	4 (80.0)	1 (20.0)
Kidney and pancreas	2 (100)	1 (0.7)	1 (1.3)	2 (100)	1 (50.0)	1 (50.0)	–	–	–
**Time from SOT to monomorphic PTLD–DLBCL**									
< 1 year	8 (100)	5 (62.5)	3 (37.5)	8 (100)	6 (75.0)	2 (25.0)	4 (100)	3 (75.0)	1 (25.0)
≥ 1 year	50 (100)	14 (28.0)	36 (72.0)	43 (100)	16 (37.2)	27 (62.8)	22 (100)	3 (13.6)	19 (86.4)
**Disease stage**									
I–II	20 (100)	5 (25.0)	15 (75.0)	17 (100)	7 (41.2)	10 (58.8)	12 (100)	1 (8.3)	11 (91.7)
III–IV	38 (100)	14 (36.8)	24 (63.2)	34 (100)	15 (44.1)	19 (55.9)	14 (100)	5 (35.7)	9 (64.3)
**IPI**									
Low risk	24 (100)	7 (29.2)	17 (70.8)	21 (100)	9 (42.9)	12 (57.1)	13 (100)	2 (15.4)	11 (84.6)
Intermediate risk	23 (100)	7 (30.4)	16 (69.6)	20 (100)	8 (40.0)	12 (60.0)	8 (100)	1 (12.5)	7 (87.5)
High risk	11 (100)	5 (45.5)	6 (54.5)	10 (100)	5 (50.0)	5 (50.0)	5 (100)	3 (60.0)	2 (40.0)
**Extranodal involvement**									
0 or 1	34 (100)	9 (26.5)	25 (73.5)	29 (100)	10 (34.5)	19 (65.5)	15 (100)	2 (13.3)	13 (86.7)
≥ 2	24 (100)	10 (41.7)	14 (58.3)	22 (100)	12 (54.5)	10 (45.5)	11 (100)	4 (36.4)	7 (63.6)
Increased LDH	32 (100)	12 (37.5)	20 (62.5)	28 (100)	12 (42.9)	16 (57.1)	13 (100)	4 (30.8)	9 (69.2)
**Chemotherapy regimen**									
R-CHOP^†^	51 (100)	13 (25.5)	38 (74.5)	45 (100)	18 (40.0)	27 (60.0)	22 (100)	3 (13.6)	19 (86.4)
R-CVP^‡^	1 (100)	1 (100)	–	1 (100)	–	1 (100)	–	–	–
Others^¶^	6 (100)	4 (80.0)	1 (20.0)	5 (100)	4 (80.0)	1 (20.0)	4 (100)	3 (75.0)	1 (25.0)

^†^Four patients were treated with CHOP.

^‡^One patient was treated with R-CVP due to a cardiac problem.

^¶^Ifosfamide, carboplatin, etoposide, and dexamethasone (ICE-Dexa) (n = 1), bendamustine and rituximab (BR) (n = 3), high dose of methotrexate and cytarabine (HD-MTX plus Ara-C) (n = 1), and no chemotherapy (n = 1); one patient underwent ileal & rectal resection.

*PTLD* post-transplantation proliferative disorder, *DLBCL* diffuse large B-cell lymphoma, *SOT* solid organ transplant, *EBV* Epstein-Barr virus, *EBER* Epstein-Barr virus-encoded small ribonucleic acids, *WB* whole blood, *IPI* international prognostic index, *LDH* lactate dehydrogenase, *R-CHOP* rituximab plus cyclophosphamide, doxorubicin, vincristine, and prednisone, *R-CVP* rituximab plus cyclophosphamide, vincristine, and prednisolone.

### Prevalence of pre-treatment EBV positivity according to clinicopathologic features

Of the patients with samples available for pre-treatment analysis (Fig. 1), positivity for EBER, WB EBV, and plasma EBV was noted in 32.8% (19/58), 43.1% (22/51), and 23.1% (6/26), respectively (Table 1). EBER, WB EBV, and plasma EBV were positive in the majority of patients who received heart transplantations and those with early-onset PTLD. Approximately two-thirds of patients with high-risk IPI scores were positive for plasma EBV. Among the 6 patients with plasma EBV (+), 5 (83.3%) had stage III–IV diseases and 4 (66.7%) had intermediate/high-risk IPI scores, extranodal involvement ≥ 2, or increased lactate dehydrogenase (LDH) levels.

### Clinical response to chemotherapy according to pre-treatment EBV status

After chemotherapy, complete response (CR), partial response (PR), stable disease (SD), and progressive disease (PD) according to the Lugano classification^24^ was achieved in 42 (72.4%), 8 (13.8%), 0 (0%), and 4 (6.9%) patients, respectively (Supplementary Table 1). A total of 54 patients with EBER-ISH, 48 patients with WB EBV PCR, and 23 patients with plasma EBV PCR were evaluable for response to chemotherapy. The rates of overall response (OR) and CR were compared according to pre-treatment EBER, WB EBV, and plasma EBV status (Table 2). Patients with EBER (+) tended to have a lower rate of OR than did those with EBER (−) (82.4% vs. 97.3%, *P* = 0.087), while the rate of CR was not significantly different according to the EBER status (70.6% vs. 81.1%, *P* = 0.389). There were no significant differences between WB EBV (+) and (−) patients in terms of OR (85.7% vs. 96.3%, *P* = 0.188) and CR (71.4% vs. 77.8%, *P* = 0.614). In contrast, patients with plasma EBV (+) had significantly lower rates of both OR (60.0% vs. 94.4%, *P* = 0.043) and CR (40.0% vs. 88.9%, *P* = 0.019) than did those with plasma EBV (−).

**Table 2:** Clinical responses according to pre-treatment EBER, whole blood EBV, or plasma EBV status.

	OR	*P*†	CR	*P*†
		0.087		0.389
EBER (+) (n = 17, %)	14 (82.4)		12 (70.6)	
EBER (−) (n = 37, %)	36 (97.3)		30 (81.1)	
		0.188		0.614
WB EBV (+) (n = 21, %)	18 (85.7)		15 (71.4)	
WB EBV (−) (n = 27, %)	26 (96.3)		21 (77.8)	
		**0.043**		**0.019**
Plasma EBV (+) (n = 5, %)	3 (60.0)		2 (40.0)	
Plasma EBV (−) (n = 18, %)	17 (94.4)		16 (88.9)	

Significant *P* values are given in bold.

^†^*P* values were estimated using the Chi-square or Fisher`s exact test.

*EBER* Epstein-Barr virus-encoded small ribonucleic acids, *WB* whole blood, *EBV* Epstein-Barr virus, *CR* complete response, *OR* overall response.

We further performed a subgroup analysis on 23 patients with evaluable disease who had both EBER and plasma EBV data. Although there were no significant differences in CR rates among patients with EBER/plasma EBV (+/+) (n = 4), EBER/plasma EBV (+/−) (n = 3), and EBER/plasma EBV (−/−) (n = 15), patients with EBER/plasma EBV (+/+) had significantly lower OR rates than those with EBER/plasma EBV (+/−) or EBER/plasma EBV (−/−) (*P* = 0.020) (Supplementary Table 2).

### Survival analysis according to pre-treatment EBV status

**Figure 2:**
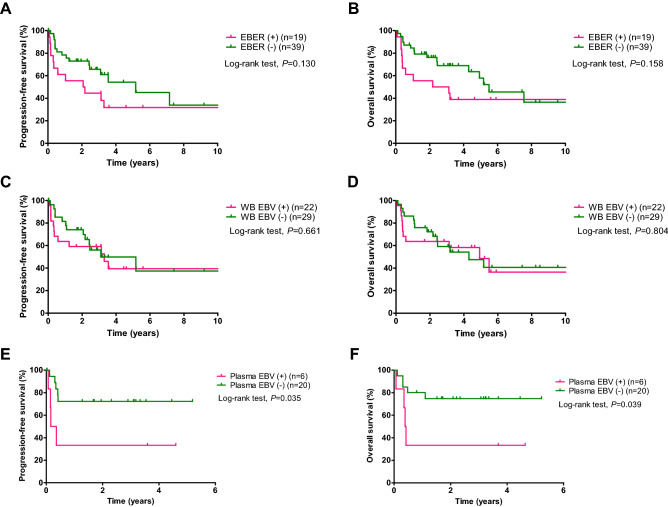
Progression-free survival and overall survival according to the pre-treatment EBER, whole blood EBV, or plasma EBV status. *EBER* Epstein-Barr virus-encoded small ribonucleic acids, *WB* whole blood, *EBV* Epstein-Barr virus.

At a median follow-up duration of 4.6 years (range 2.7–6.6), the rates of 2-year PFS and OS in the whole cohort were 68.9% and 70.7%, respectively. There were no significant differences in the PFS and OS according to the pre-treatment status of EBER or WB EBV (Fig. 2A–D). In contrast, patients with plasma EBV (+) had significantly worse PFS (33.3% vs. 75.0%, log-rank *P* = 0.035) and OS (33.3% vs. 75.0%, log-rank *P* = 0.039) than did those with plasma EBV (−) PTLD (Fig. 2E–F).

The results of univariate and multivariate analyses are shown in Table 3. In univariate analysis, Old age (> 60 years), extranodal involvement ≥ 2, increased LDH, intermediate or high-risk IPI score, and plasma EBV DNA (+) were associated with poor PFS and OS (*P* < 0.1). In multivariate analysis with IPI scores, plasma EBV DNA (+) remained as a significant factor for poor PFS (HR 4.92, 95% confidence interval [CI] 1.22–19.86, *P* = 0.025) and poor OS (HR 4.48, 95% CI 1.14–17.63, *P* = 0.032).

**Table 3:** Univariate and multivariate analysis for progression-free survival and overall survival.

	Progression-free survival				Overall survival			
	Univariate	*P*†	Multivariate*	*P*†	Univariate	*P*†	Multivariate*	*P*†
Age at PTLD–DLBCL (> 60 years vs. $\le \hspace{0.17em}$60 years) (n = 18 vs. 40)	1.93 (0.91–4.09)	0.085			1.94 (0.91–4.15)	0.088		
Sex (male vs. female) (n = 39 vs. 19)	0.78 (0.35–1.74)	0.535			0.69 (0.31–1.56)	0.371		
Extranodal involvement ($\ge$ 2 vs. 0–1) (n = 24 vs. 34)	2.08 (0.98–4.39)	0.055			1.80 (0.84–3.84)	0.129		
Increased LDH (yes vs. no) (n = 32 vs. 26)	2.78 (1.22–6.33)	**0.015**			3.26 (1.37–7.77)	**0.008**		
Stage (III–IV vs. I–II) (n = 38 vs. 20)	1.99 (0.80–4.91)	0.137			1.48 (0.62–3.50)	0.375		
ECOG PS ($\ge$ 2 vs. 0–1) (n = 7 vs. 51)	1.62 (0.61–4.27)	0.331			1.38 (0.47–3.99)	0.558		
Time from SOT to monomorphic PTLD–DLBCL (> 1 year vs. $\le$ 1 year) (n = 50 vs. 8)	1.46 (0.44–4.86)	0.539			1.65 (0.50–5.51)	0.414		
**Transplant type**								
Kidney (n = 22)	1				1			
Liver (n = 27)	1.17 (0.52–2.64)	0.709			0.95 (0.41–2.17)	0.896		
Heart (n = 7)	1.86 (0.58–5.96)	0.297			1.88 (0.58–6.04)	0.290		
Kidney and Pancreas (n = 2)	NA	0.982			NA	0.983		
**IPI**								
Low (n = 24)	1		1		1		1	
Intermediate (n = 23)	2.44 (0.96–6.25)	0.062	3.11 (1.12–8.58)	**0.029**	2.28 (0.90–5.81)	0.083	2.73 (1.02–7.29)	**0.045**
High (n = 11)	3.58 (1.33–9.63)	**0.012**	4.00 (1.42–11.25)	**0.009**	3.03 (1.10–8.39)	**0.032**	3.19 (1.12–9.05)	**0.029**
EBER ((+) vs. (−)) (n = 19 vs. 39)	1.77 (0.84–3.75)	0.135			1.73 (0.80–3.73)	0.164		
WB EBV ((+) vs. (−)) (n = 22 vs. 29)	1.23 (0.56–2.71)	0.606			1.15 (0.52–2.54)	0.735		
Plasma EBV ((+) vs. (−)) (n = 6 vs. 20)	3.78 (1.00–14.30)	**0.050**	4.92 (1.22–19.86)	**0.025**	3.81 (1.02–14.30)	**0.047**	4.48 (1.14–17.63)	**0.032**

Significant *P* values are given in bold.

^†^*P* values were estimated using the Cox proportional regression model.

*In the multivariate analysis, variables exhibiting a potential association with survival (*P* < 0.1) in the univariate analysis, along with EBER, WB EBV, and plasma EBV were included. IPI was included in multivariate analysis as a categorical variable; low (0–1 risk), intermediate (2–3 risks), and high (4–5 risks).

*PTLD* post-transplantation proliferative disorder, *DLBCL* diffuse large B-cell lymphoma, *SOT* solid organ transplant, *NA* not applicable, *EBER* Epstein-Barr virus-encoded small ribonucleic acids, *WB* whole blood, *EBV* Epstein-Barr virus, *IPI* international prognostic index.

**Figure 3:**
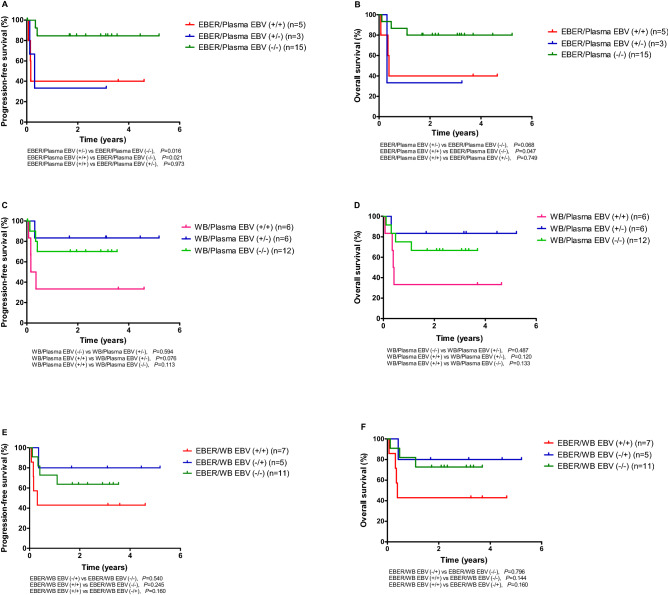
Progression-free survival and overall survival according to the pre-treatment EBER, whole blood EBV, or plasma EBV status in patients with results from all three tests (n = 24). *EBER* Epstein-Barr virus-encoded small ribonucleic acids, *WB* whole blood, *EBV* Epstein-Barr virus. *There was no patient with WB/plasma EBV (−/ +). In addition, there was only one patient each with EBER/plasma EBV (−/ +) or EBER/WB EBV (+ /−), and were excluded from this analysis.

We further performed a subgroup analysis of 24 patients in whom the results of all three tests—EBER-ISH, WB EBV DNA PCR, plasma EBV DNA PCR—were available (Fig. 3). Patients with EBER/plasma (+/−) had worse PFS (*P* = 0.016) and OS (*P* = 0.068) compared with those with EBER/plasma (−/−). There were no significant differences in PFS (*P* = 0.973) and OS (*P* = 0.749) between patients with EBER/plasma EBV (+/+) and those with EBER/plasma (+/−). Patients with EBER/plasma EBV (+/+) had significantly worse PFS (*P* = 0.021) and OS (*P* = 0.047) compared with those with EBER/plasma EBV (−/−). Whereas there were no significant differences in PFS (*P* = 0.594) and OS (*P* = 0.487) between patients with WB/plasma EBV (+/−) and those with WB/plasma EBV (−/−), patients with WB/plasma EBV (+/+) tended to have worse PFS (*P* = 0.076) and OS (*P* = 0.120) compared with those with WB/plasma EBV (+/−). Moreover, there were no significant differences in PFS (*P* = 0.540 and 0.160) and OS (*P* = 0.796 and 0.160) between patients with EBER/WB EBV (−/+) and those with EBER/WB EBV (−/−) and between patients with EBER/WB EBV (+/+) and those with EBER/WB EBV (−/+), respectively.

### Clinical response and survival analysis according to pre-treatment EBV status in patients treated with R-CHOP or CHOP

Among the 58 patients, 51 patients were treated with R-CHOP or CHOP. Response with chemotherapy was evaluable in 48 patients; EBER-ISH (n = 48), WB EBV PCR (n = 42), and plasma EBV PCR (n = 19), while two patients died of treatment-related complications during cycle 1 or 2 of R-CHOP and the other patient was lost to follow-up after cycle 1 of R-CHOP. There were no significant differences in OR and CR rates according to pre-treatment EBER, WB EBV, and plasma EBV status, respectively (Supplementary Table 3). There were no significant differences in the PFS and OS according to the pre-treatment status of EBER or WB EBV (Supplementary Fig. 2A–D). However, patients with plasma EBV (+) had a trend towards poor PFS (*P* = 0.060) and significantly worse OS (*P* = 0.041) compared to those with plasma EBV (-) (Supplementary Fig. 2E–F).

## Discussion

We investigated the prognostic value of pre-treatment EBV status in the tissue and blood (WB and plasma) in patients who developed monomorphic PTLD–DLBCL after solid organ transplantation. Our results suggest that the plasma EBV DNA may be more prognostic for survival than tissue EBER or WB EBV DNA in monomorphic PTLD–DLBCL following solid organ transplantation. The rates of OR and CR were significantly lower in patients with plasma EBV positivity than in those without. Moreover, plasma EBV positivity was significantly associated with poor PFS and OS and remained as a significant marker of poor prognosis in multivariate analysis. In contrast, EBER and WB EBV status were not significantly associated with OR, CR, PFS, or OS.

Following primary infection, EBV persists mainly in memory B cells, T cells, or NK cells^25,26^. Prolonged immunosuppression followed by solid organ transplantation induces the deficiency of EBV-specific cytotoxic T lymphocytes, which results in uncontrolled proliferation of EBV-infected lymphocytes leading to the development of PTLD^25^. After EBV-driven lymphoproliferation exceeds a certain level, EBV-infected cells are released from lymphoid tissues to peripheral blood^25,27^. Considering such pathogenesis of PTLD, the blood EBV DNA may be a better marker than tissue EBER-ISH for assessing disease severity and disease burden.

There are three main approaches for the quantification of EBV DNA in the peripheral blood: WB, peripheral blood mononuclear cells (PBMC), and plasma. Growing evidence supports the notion that cell-free plasma EBV DNA is a better marker than cellular (WB or PBMC) EBV DNA^17,28,29^. Considering that low-level reactivation of EBV can occur in the lymphoid tissues of immunocompromised patients without PTLD^17,28,30^, WB EBV DNA (including cellular EBV) is more likely to be detectable in such patients. Indeed, in a previous prospective trial, positive rate of EBV DNA in transplant patients without PTLD was higher in WB samples compared with plasma samples (42% vs. 3%)^28^. Furthermore, blood EBV DNA must be quantified per unit of total DNA, and this can vary according to leukocyte counts in the sample. In addition, the number of circulating memory B cells, which are the main reservoirs of EBV, is unpredictable in WB and can introduce errors. There are also technical issues with quantitative PCR when processing leukocyte DNA^25,31^. In our subgroup analysis, there were no patients with WB/plasma EBV (−/+), while there were six patients with WB/plasma EBV (+/−) who showed favorable survival and achieved an OR rate of 100%. Taken together, the plasma may be the most appropriate sample to assess the prognosis of PTLD.

Previous studies have reported conflicting results regarding the prognostic value of EBV status in PTLD, and its impact on survival is still not clear^11–17^. However, most of these studies evaluated the prognostic value of EBV status in a heterogeneous group of patients with PTLD, with variations in histologic subtypes (early lesions vs. polymorphic vs. monomorphic PTLD), treatment (reduction of immunosuppressive agents vs. chemotherapy), and transplantation type. Furthermore, the EBV status was determined only by tissue EBER-ISH. In our study, the prognostic value of EBV status was evaluated in a homogeneous group of patients with monomorphic PTLD–DLBCL treated with chemotherapy. As a result, we found that EBER positivity was not significantly associated with PFS and OS. This finding is in line with the results of a previous phase 2 trial on a relatively homogeneous group of patients with B-cell PTLD treated with rituximab and chemotherapy, which showed that EBER status was not significantly associated with survival^6^. Nevertheless, our subgroup analysis of 24 patients with all three test results (EBER-ISH, WB EBV DNA PCR, and plasma EBV DNA PCR) showed that the EBER status could also have some importance among patients with plasma EBV negativity, as patients with EBER/plasma (+/−) had worse PFS (*P* = 0.016) and OS (*P* = 0.068) than did those with EBER/plasma (−/−). These results need to be interpreted with caution as they were derived from a subgroup analysis with a small sample size. Also, there may be some variations in the amount of EBV DNA and the intensity of EBER staining under a certain level in lymphoid tissues, which may also contribute to the discrepant associations between EBER and prognosis.

With respect to the type of transplantation, we found that patients who underwent heart transplantation were more likely to be plasma EBV (+) compared with patients who underwent other transplantations. The level of LDH was also higher in patients with plasma EBV (+) than in patients with plasma EBV (−), while it was not significantly associated with the EBER or WB EBV status (Supplementary Fig. 3). Serum LDH level may be associated with tumor microenvironment including various immune cells as well as tumor burden and aggressiveness in lymphoid malignancy^32^, and its negative prognostic value is well-documented in patients with Hodgkin or non-Hodgkin lymphoma^32–34^.

An issue regarding the evaluation of the blood EBV DNA status in patients with EBER (−) PTLD–DLBCL may be raised. However, among the 39 patients with EBER (−) PTLD–DLBCL in this study, most of the patients had either WB EBV (−) or plasma EBV (−), and blood EBV DNA status was unknown in only three (7.7%) patients. In the subgroup analysis of 24 patients with all three test results, five had EBER/WB (−/+) and one had EBER/plasma (−/+). WB EBV DNA includes intracellular EBV DNA not associated with tumors, which can be detected in the immunosuppressed state^17^, and the possibility of false positivity can be considered in EBER (−) PTLD^28^. In one patient with EBER/plasma (−/+), EBER-ISH was performed in a specimen derived from the core needle biopsy raising the possibility of false negativity of the EBER status as there can be heterogeneity in EBER status in tumors^35,36^. Heterogeneous EBER expression was initially reported by Hummel et al.^35^ and EBER (+) and EBER (−) cells were evenly intermixed in four (10.3%) out of 39 patients who were classified into EBER (+)^36^. The clinical implication of blood EBV DNA (+) in EBER (−) PTLD–DLBCL should be elucidated in large-sized studies.

There are some limitations to this study. The sample size of patients who underwent all three tests (EBER-ISH, WB EBV DNA PCR, plasma EBV DNA PCR) was relatively small (n = 24), as it is not mandatory to perform both WB and plasma EBV PCR in all patients. The sample size of six patients with positive plasma EBV was too small and too confounded. The detection kit for blood EBV PCR used at our center has changed over time, thus limiting the unity of the EBV status results. Nevertheless, to the best of our knowledge, this study is the first to evaluate the prognostic value of blood EBV status and comprehensively compare the results of EBER-ISH, WB EBV PCR, and plasma EBV PCR tests in a homogeneous group of patients with monomorphic PTLD–DLBCL. Although the sample size was small, we found significant differences in OR and CR rates, and the PFS and OS curves also significantly diverged according to plasma EBV status.

In conclusion, our study showed that plasma might be a more appropriate sample than tissue EBER-ISH and WB for evaluating the EBV DNA loads in patients with monomorphic PTLD–DLBCL after solid organ transplantation. Plasma EBV DNA measured before treatment had significant prognostic values and may be used to indicate the need for additional treatments in patients with monomorphic PTLD–DLBCL.

## Methods

### Patients

Data were retrieved from a prospectively collected database in which 70 adult patients with newly diagnosed monomorphic PTLD after solid organ transplantation at Asan Medical Center (Seoul, South Korea) were consecutively enrolled between December 2004 and June 2019. The diagnosis of monomorphic PTLD was based on pathological examination in accordance with the World Health Organization classification^1,2^. The patients with monomorphic PTLD–DLBCL were only included in the final analysis. Baseline data included laboratory tests (complete blood count, liver and kidney function test, LDH), EBV serostatus at the time of transplantation, bone marrow aspiration and biopsy, computed tomography (CT) scans, and whole-body positron emission tomography-CT (PET-CT) scans. The EBV status prior to treatment was assessed (Fig. 1 and Supplementary Fig. 1) by EBER-ISH in tissue samples (n = 58), EBV DNA real-time quantitative PCR in WB samples (n = 51), and/or EBV DNA real-time quantitative PCR in plasma samples (n = 26).

Following the completion of planned treatment for monomorphic PTLD–DLBCL^37–40^, the treatment response in each patient was assessed by CT scans and PET-CT scans at least four weeks after the last treatment cycle according to the Lugano classification^24^. This study was approved by the Institutional Review Board at Asan Medical Center (IRB No. 2019-1476) and has been confirmed for waiver of informed consent because of the retrospective nature. This study was conducted in accordance with the Declaration of Helsinki and Good Clinical Practice.

### In situ hybridization (ISH) for EBV-encoded RNA

**Figure 4:**
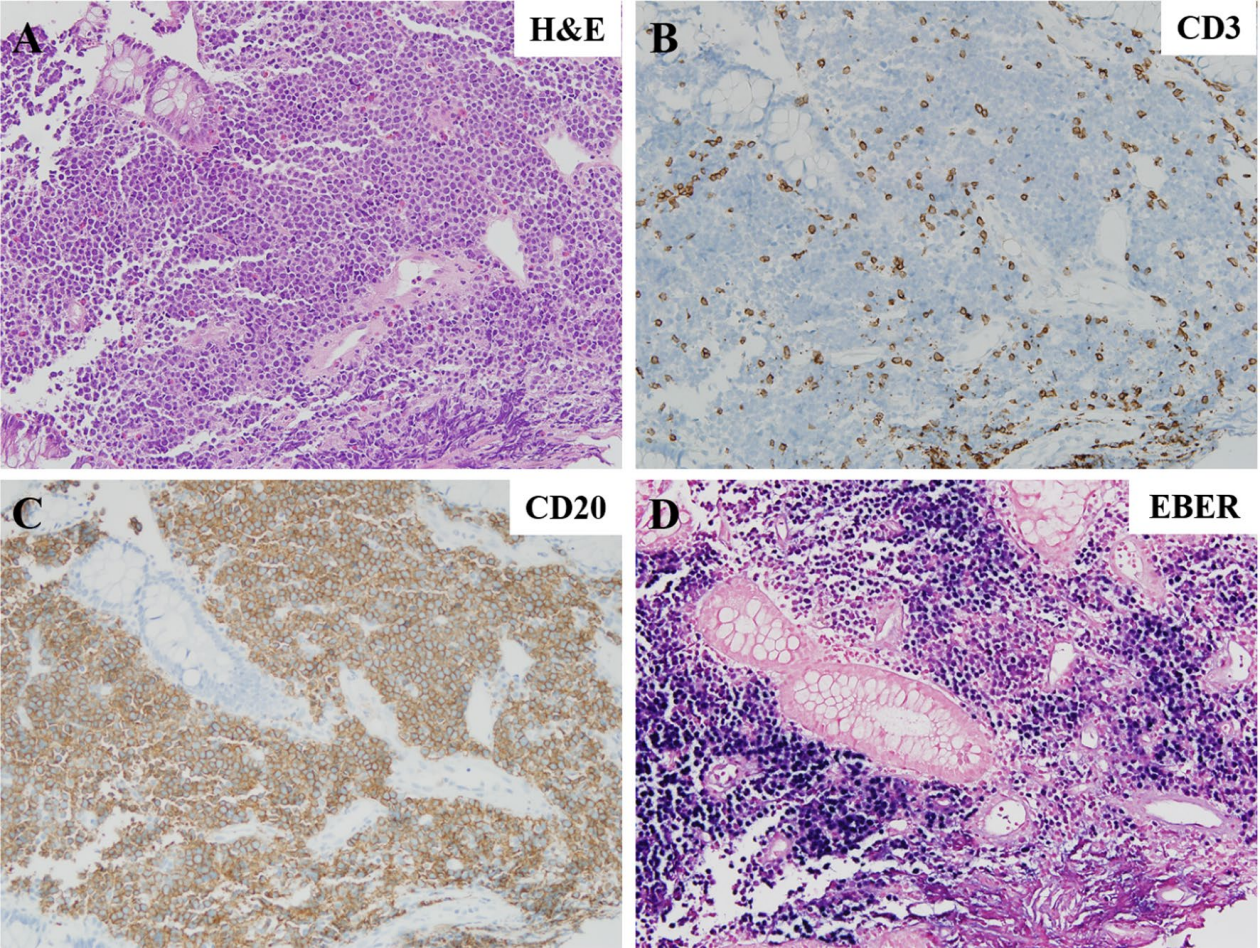
Histologic examination of a monomorphic PTLD–DLBCL involving terminal ileum (**A**) hematoxylin and eosin, original magnification ×200, (**B**) tumor cells were negative for CD3, (**C**) while positive for CD20 and (**D**) EBER. *PTLD* post-transplantation proliferative disorder, *DLBCL* diffuse large B-cell lymphoma, *EBER* Epstein-Barr Virus-encoded small ribonucleic acid.

Biopsy samples (4-μm thick) were mounted on glass slides to detect EBER using automated ISH (BENCHMARK, VENTANA MEDICAL SYSTEM, Oro Valley, AZ, USA), previously described^21^. Briefly, tissue samples were treated with ISH protease 2 (catalog no. 780-4147; VENTANA MEDICAL SYSTEM) and labeled with an EBER-specific probe (catalog no. 780-2842; VENTANA MEDICAL SYSTEM), which was detected using the Inform Probes iView Blue V3 detection kit (VENTANA MEDICAL SYSTEM)^21^. A sample was considered EBV (+) if definitive tumor cells were labeled with the EBER probe, and EBV (−) if the EBER-ISH was negative in tumor cells or positive only in bystander cells. The EBER staining patterns were interpreted by a hematopathologist who was blinded to the clinical outcomes of the patients. Histologic findings from a representative case is shown in Fig. 4.

### Evaluation of the EBV status in whole blood (WB) and plasma

The EBV DNA viral load was measured in WB or plasma samples by real-time quantitative PCR before the planned treatment, where available. The EBV DNA viral load was measured only in WB samples between 2004 and 2014^21^. Thereafter, the measurement was performed in both WB and plasma samples between September 2014 and September 2018, and only in plasma samples between October 2018 and June 2019. Peripheral blood samples were collected in EDTA-containing tubes. DNA was manually extracted using the QIAAMP DNA mini kit (QIAGEN, Hilden, Germany) from blood samples (200 μL) according to the manufacturer’s instructions^21^. The extracted DNA was eluted with 50 μL of elution buffer and immediately quantified. The EBV DNA load was quantified using the ARTUS EBV Light Cycler Kit (QIAGEN) that ran on the Light Cycler platform, version 2.0 (ROCHE DIAGNOSTICS International AG, Switzerland). The targeted gene amplified in the ARTUS kit was *EBNA1*. In this kit, the detectable values had a cutoff of ≥ 2.66 log copies/mL (1000 copies/mL). From September 2018, the ABBOTT REAL TIME EBV kit (ABBOTT MOLECULAR Inc., Des Plaines, IL, USA) targeting the *BLLF1* gene was used, and the cutoff value for plasma samples was 1.60 log IU/mL (281.8 copies/mL).

### Statistical analysis

The chi-squared test or Fisher’s exact test was used to compare categorical variables, and the Mann–Whitney *U* test was used to compare continuous variables^21^. Overall survival (OS) was defined as the time from the initial date of treatment to the date of death from any cause or to the date of the last follow-up visit for alive patients. Progression-free survival (PFS) was defined as the time from the initial date of treatment to the date of relapse, progression, or death from any cause. Survival rates were estimated using the Kaplan–Meier method, and comparisons were performed using the log-rank test^21^. The Cox proportional hazard regression model was used to assess the prognostic impact of EBER, WB EBV, and plasma EBV status on PFS and OS. In the multivariate analysis, variables exhibiting a potential association with survival (*P* < 0.1) in the univariate analysis, along with EBER, WB EBV, and plasma EBV were included. IPI was included in multivariate analysis as a categorical variable; low (0–1 risk), intermediate (2–3 risks), and high (4–5 risks). Two-sided *P* values smaller than 0.05 were considered statistically significant, and all statistical analyses were performed using IBM SPSS Statistics for Windows, Version 21.0 (IBM Corp., Armonk, NY, USA).

## Supplementary Information

Supplementary Information.

## Data Availability

The datasets generated during and/or analyzed during the current study are available from the corresponding author (DHY and CS) on reasonable request. The data are not publicly available due to them containing information that could compromise research participant privacy/consent.
