# Supplementary material for: Reappraisal of the prognostic value of Epstein-Barr virus status in monomorphic post-transplantation lymphoproliferative disorders–diffuse large B-cell lymphoma
Source: Sci Rep. 2021 Feb 3;11:2880. doi: 10.1038/s41598-021-82534-4 (PMC7859229; doi:10.1038/s41598-021-82534-4)
Supplement: Supplementary file 1 — Supplementary Information. [file 41598_2021_82534_MOESM1_ESM.docx]

**Supplementary Table 1. Clinical responses according to the pre-treatment EBER, whole blood EBV, or plasma EBV status**

|  | **Total patients**  **(n=58)** | **EBER (+)**  **(n=19)** | **EBER (-)**  **(n=39)** | **WB EBV (+) (n=22)** | **WB EBV (-) (n=29)** | **Plasma EBV (+) (n=6)** | **Plasma EBV (-) (n=20)** |
| --- | --- | --- | --- | --- | --- | --- | --- |
| **CR** | 42 (72.4) | 12 (63.2) | 30 (76.9) | 15 (68.2) | 21 (72.4) | 2 (33.3) | 16 (80.0) |
| **PR** | 8 (13.8) | 2 (10.5) | 6 (15.4) | 3 (13.6) | 5 (17.2) | 1 (16.7) | 1 (5.0) |
| **PD** | 4 (6.9) | 3 (15.8) | 1 (2.6) | 3 (13.6) | 1 (3.4) | 2 (33.3) | 1 (5.0) |
| **NE^*^** | 4 (6.9) | 2 (10.5) | 2 (5.1) | 1 (4.5) | 2 (6.9) | 1 (16.7) | 2 (10.0) |

Values are presented as n (%).

*Expired during treatment of complication (n = 2) and no chemotherapy (n = 1); one patient underwent ileal & rectal resection, and was lost to follow-up (n = 1).

Abbreviations: EBV; Epstein-Barr virus, EBER; Epstein-Barr virus-encoded small RNAs, WB; whole blood, CR; complete response, PR; partial response, PD; progressive disease, NE; not evaluable

**Supplementary Table 2.** **Clinical responses according to pre-treatment EBER and plasma EBV status**

|  | **EBER/plasma EBV (+/+) (n = 4, %)** | **EBER/plasma EBV (+/-) (n = 3, %)** | **EBER/plasma EBV (-/-) (n = 15, %)** | ***P***† |
| --- | --- | --- | --- | --- |
| **OR rates** | 2 (50.0) | 2 (66.7) | 15 (100) | **0.020** |
| **CR rates** | 2 (50.0) | 2 (66.7) | 14 (93.3) | 0.104 |

Abbreviations: EBER, Epstein-Barr virus-encoded small ribonucleic acids; EBV, Epstein-Barr virus; CR, complete response; OR, overall response.

*Only one patient with EBER/plasma EBV (-/+) was excluded from this analysis.

†: *P* values were estimated using the Chi-square or Fisher`s exact test.

**Supplementary Table 3.** **Clinical responses according to pre-treatment EBER, whole blood EBV, or plasma EBV status in evaluable patients treated with R-CHOP or CHOP**

|  | **OR** | ***P***† | **CR** | ***P***† |
| --- | --- | --- | --- | --- |
|  |  | 0.441 |  | 0.671 |
| **EBER (+) (n = 12, %)** | 11 (91.7) |  | 9 (75.0) |  |
| **EBER (−) (n = 36, %)** | 35 (97.2) |  | 30 (83.3) |  |
|  |  | 1.000 |  | 1.000 |
| **WB EBV (+) (n = 17, %)** | 16 (94.1) |  | 13 (76.5) |  |
| **WB EBV (−) (n = 25, %)** | 24 (96.0) |  | 20 (80.0) |  |
|  |  | 1.000 |  | 0.298 |
| **Plasma EBV (+) (n = 2, %)** | 2 (100) |  | 1 (50.0) |  |
| **Plasma EBV (−) (n = 17, %)** | 16 (94.1) |  | 15 (88.2) |  |

†: *P* values were estimated using the Chi-square or Fisher`s exact test.

Abbreviations: EBER, Epstein-Barr virus-encoded small ribonucleic acids; WB, whole blood; EBV, Epstein-Barr virus; CR, complete response; OR, overall response.

**Supplementary Figure 1. Data availability according to different tests for evaluating EBV status**


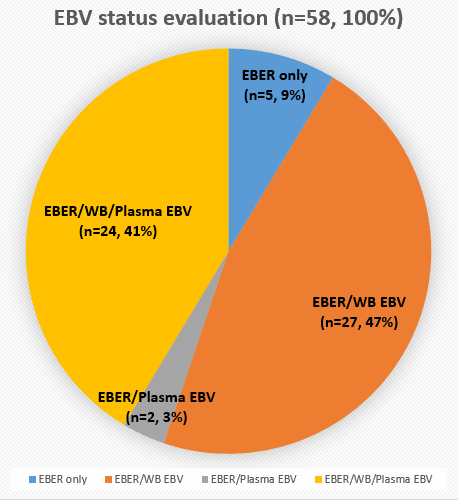


Abbreviations: EBER, Epstein-Barr virus-encoded small ribonucleic acids; WB, whole blood; EBV, Epstein-Barr virus.

**Supplementary Figure 2. Progression-free survival and overall survival according to the pre-treatment EBER, whole blood EBV, or plasma EBV status in patients treated with R-CHOP or CHOP.**

Abbreviations: EBER; Epstein-Barr virus-encoded small ribonucleic acids, WB; whole blood, EBV; Epstein-Barr virus

**Supplementary Figure 3. Lactate dehydrogenase levels according to pre-treatment EBER-ISH, whole blood EBV, or plasma EBV status**
